# What Is the Evidence that Parkinson’s Disease Is a Prion Disorder, Which Originates in the Gut?

**DOI:** 10.3390/ijms19113573

**Published:** 2018-11-12

**Authors:** Małgorzata Kujawska, Jadwiga Jodynis-Liebert

**Affiliations:** Department of Toxicology, Poznan University of Medical Sciences, 30 Dojazd Str., 60-631 Poznań, Poland; liebert@ump.edu.pl

**Keywords:** enteric nervous system, dysbiosis, Braak’s staging system

## Abstract

Parkinson’s disease (PD) is a neurodegenerative disorder resulting from degeneration of dopaminergic neurons in the substantia nigra pars compacta (SNpc). PD is characterized by motor dysfunctions as well as gastrointestinal symptoms and mental impairment. The pathological hallmark of PD is an accumulation of misfolded α-synuclein aggregates within the brain. The etiology of PD and related synucleinopathy is poorly understood, but recently, the hypothesis that α-synuclein pathology spreads in a prion-like fashion originating in the gut has gained much scientific attention. A crucial clue was the appearance of constipation before the onset of motor symptoms, gut dysbiosis and synucleinopathy in PD patients. Another line of evidence, demonstrating accumulation of α-synuclein within the peripheral autonomic nervous system (PANS), including the enteric nervous system (ENS), and the dorsal motor nucleus of the vagus (DMV) support the concept that α-synuclein can spread from the ENS to the brain by the vagus nerve. The decreased risk of PD following truncal vagotomy supports this. The convincing evidence of the prion-like behavior of α-synuclein came from postmortem observations that pathological α-synuclein inclusions appeared in healthy grafted neurons. In this review, we summarize the available data from human subjects’ research and animal experiments, which seem to be the most suggestive for explaining the hypotheses.

## 1. Introduction

Parkinson’s disease (PD) is a chronic human neurodegenerative disorder, which is characterized by motor dysfunction including tremors, bradykinesia, rigidity and postural instability. The neuropathology of the symptoms is associated with a severe loss of dopaminergic (DAergic) neurons in the substantia nigra pars compacta (SNpc) and progressive accumulation of Lewy pathology (LP) in the form of Lewy bodies (LBs) and Lewy neurites (LNs), mainly composed of misfolded α-synuclein [1].

In addition to the motor symptomatology, nonmotor manifestations such as gastrointestinal dysfunction, including constipation, delayed gastric emptying, hypersalivation, dysphagia and nausea as well as olfactory impairment, depression, and sleep disturbances appear throughout disease stages in PD. It has been reported that the nonmotor symptoms, mainly constipation and olfactory deficit, predate the motor ones, sometimes by up to ten years [2].

A growing body of evidence highlights the relevance of constipation, with prevalence in PD patients ranging from 24.6% to 63.0%; to be not only the symptom but also to play a crucial role in the development of pathophysiological changes underlying motor impairment [3]. The slow colonic motility depending on intrinsic circuits in the enteric nervous system (ENS) appears to be the primary cause of the anomaly in gut functionality in PD. The ENS consists of two plexuses, myenteric (or Auerbach’s plexus) and submucosal (or Meissner plexus) located within the walls of the gut and controls gastrointestinal function independently from the central nervous system (CNS) [4]. Braak et al. [5] based on their neuropathological findings have suggested that the earliest neuropathological features of PD occur in the ENS, olfactory bulbs, and lower brainstem, with particular reference to the dorsal motor nucleus of the vagus (DMV). Furthermore, a pathological hallmark of the disease, LBs, in the olfactory epithelium [6] and in both submucosal and mucosal plexuses of the gut, from the oesophagus to the rectal end in PD patients, has been reported to reflect the preclinical stage of PD [7,8]. Braak and Tredici [9] have formulated the hypothesis that the abnormal α-synuclein deposition begins in the olfactory bulbs and/or in the ENS, due to the action of an environmental agent or pathogen, and then proceeds towards the SNpc and further sites in the CNS.

Based on the above it is suggested that in the very early stages of PD, the first event of α-synuclein misfolding occurs in the ENS and then spreads transneuronally in a prion-like fashion through the gut-brain ascending pathways to the CNS and finally causes the degeneration of DAergic neurons in the SNpc.

## 2. The Hypothesis of Prion-Like Propagation of A-Synuclein within the Gut-Brain Axis

Prion stands for ‘proteinaceous infectious particle’ which can self-transform its shape and propagate and thus can be transmitted from cell-to-cell, between individuals and even different species. Emerging evidence suggests that misfolded α-synuclein behaves in a prion-like fashion, underlying its intercellular transfer and interregional transport, that contributes to the neuropathology of Parkinson’s disease. Of note, epidemiological studies conducted so far do not support the hypothesis that α-synucleinopathy might be communicable [10].

A starting point of the hypothesis came from independent postmortem observations that embryonic mesencephalic “healthy” neurons grafted into the brains of PD patients acquired LBs several years after the transplantation [11,12,13]. Numerous in vitro experiments have demonstrated that cultured neurons can both secrete and take α-synuclein up from the extracellular environment, which supports the idea of its transcellular transmission in a prion-like manner [14,15,16]. Based on the results of the studies, a hypothetical model of α-synuclein cell-to-cell transmission has been developed that assumes a prion-like mechanism, by which native protein with α-helical structure adopts a toxic form with a β-sheet structure, triggering the misfolding of further native protein in a self-perpetuating process [17]. Thereby, the abnormal protein acts as a seed or template for misfolding normal α-synuclein. However, whether the seeds are constantly formed and actively removed throughout life, or their formation is rather a rare event initiating disease, is still unsolved. The stabilized β-sheet conformations aggregate in neurons into toxic assemblies composed of fibrillar, phosphorylated and ubiquitinated α-synuclein—the main component of LBs and LNs. The export of intraneuronal α-synuclein assemblies into the extracellular space and then its uptake by neighboring neurons or astrocytes are crucial for the prion hypothesis of PD. It is suggested that neurons containing LBs could release amyloid aggregates and seeds them into the extracellular milieu by non-classical exocytosis, in which aberrant α-synuclein is targeted by a ubiquitin-specific protease or via exosomes, as well as after cell injury or death. Exosomes, microvesicles and tunneling nanotubes (TNTs) are proposed, in turn, to participate in the transfer of α-synuclein from one cell to another. The extracellular α-synuclein can then undergo internalization by endocytosis through either neighbor neurons or glial cells. The uptake of exogenous seeds directly promotes the conversion of α-synuclein monomers in the recipient cell into aggregates and fibrils, and new LBs are formed as a result [18,19]. Besides the mentioned above TNTs several other biomolecules, such as the ubiquitin-specific protease 19, heat shock protein 70, and lymphocyte-activation gene 3 (LAG3) are suggested to be involved in the prion-like spread of α-synuclein [20]. It should be noted that cross-sectional histopathological examinations have revealed that proteinaceous lesions appear and spread in disease-specific spatiotemporal patterns [19].

The gut-brain axis model facilitates a bidirectional network communication through biochemical signaling between the ENS and CNS [21]. This model has been reported to comprise the CNS, the neuroimmune and neuroendocrine systems, the parasympathetic and sympathetic sections of the autonomic nervous system and the gut microbiota [22]. In the context of Parkinson’s disease, a putative role of intestinal microbiota in regulating the gut-brain axis has recently been evoked. Several pathways have been demonstrated to be involved in the gut microbiota–brain communication, including neural connections (vagus nerve), neuroactive mediators produced by commensals bacteria during their metabolism, mainly short chain fatty acids (SCFAs), proinflammatory cytokines, and tryptophan metabolism as well as the effects of intestinal microbiota on gut function, metabolism, and immune system of the host [23,24]. Results from experiments on animals have suggested that α-synuclein can spread from the ENS to the brain by the vagus nerve [25,26,27]. The hypothesis is supported by the data about the decreased risk of developing PD in patients who underwent a full truncal vagotomy [28]. Research on the intestinal tissue of PD patients has revealed the altered concentrations of SCAFs [29] as well as increased intestinal permeability [30] that correlates with changes in colonic microbiota. In addition, analysis of mucosal biopsies and faecal samples provides evidence that the first event of α-synuclein misfolding in the gastrointestinal (GI) tract does not necessarily need a pathogenic or environmental stimulus since proinflammatory dysbiosis present in the gut of PD patients could trigger inflammation-induced α-synucleinopathy and the development of PD pathology [31].

**Figure 1:**
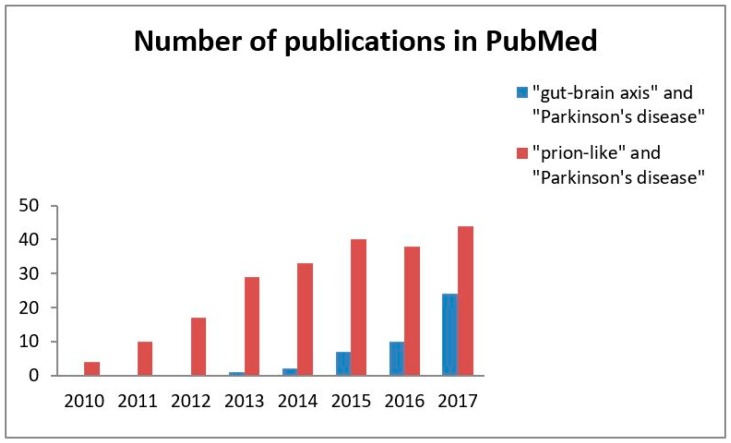
A number of publications on the concept of the gut-brain axis and α-synuclein as a prion-like protein in Parkinson’s disease. The search was performed for each entire year (2010–2017). The values are the crude total number of articles published in English per year according to PubMed.

The scientific community is becoming increasingly aware of the importance of the hypothesis that the α-synuclein aggregation spreads via a prion-like mechanism originating in the gut. Searching PubMed for references for this review has revealed a steadily increasing number of articles devoted to this topic (Figure 1). The present review is intended to provide a comprehensive overview of current human and experimental evidence on the hypothesis that α-synuclein may be transmitted in a prion-like manner within the gut-brain axis.

## 3. Evidence in PD Patients

Over the last decade, the connection between the intestinal environment and the CNS has attracted the interest of researchers with a great emphasis on the role of intestinal microbiota in regulating the gut-brain axis [20,24,32]. Given the evidence in human subjects, summarized in Table 1, it is quite likely that gut microbes contribute to the pathogenesis of PD. In this section, we focus on the potential of intestinal health impairments to the development of the neurodegenerative condition.

Recent studies on the gut microbiota have demonstrated gut dysbiosis [29,30,31,33,34,35,36,37,38] and/or small intestinal overgrowth (SIBO) [39,40,41,42] in PD patients, which correlated well with the severity of motor dysfunction [34,39,40] or with the GI impairment, such as constipation [33,34], bloating and flatulence [39], increased gut permeability [30,33] and inflammation [30]. A systematic review by Gerhardt and Mohajeri [43] have summarized the findings on the composition of fecal or intestinal bacteria populations in PD patients published between 1 January 2013 and 31 December 2017 showing an increase in *Lactobacillus*, *Bifidobacterium*, Verrucomicrobiaceae, and *Akkermansia* and a decrease in *Faecalibacterium* spp., *Coprococcus* spp., *Blautia* spp., *Prevotella* spp. and Prevotellaceae.

Since enteric bacteria possess the capacity to produce various neuroactive molecules, such as serotonin, catecholamines, glutamate, gamma-aminobutyric acid (GABA) and SCFAs interacting with the intrinsic enteric neurons of the ENS and extrinsic afferent neurons of the CNS, it has been hypothesized that they are able to influence GI motility as well as CNS functions [44]. Intestinal microbes are the source of SCFAs such as acetic acid, propionic acid, and butyric acid, which are produced during the fermentation process. These molecules, in particular butyrate, are known to play an important role in maintaining the colonic epithelium integrity through the regulation of tight junction proteins, including zona occludens proteins and occludin [45]. Importantly, the decreased expression of occludin has been demonstrated in colonic samples from PD patients, in which LP in the submucosa has been detected [46]. The increased permeability has been demonstrated to enable translocation of bacteria and endotoxin, subsequently to trigger an inflammatory cascade leading to local and/or systemic inflammation as a consequence [45]. Moreover, the gut-derived inflammation has been suggested to be involved in PD pathology [32,47]. On the other hand, butyric acid was reported to have also a beneficial effect against constipation by increasing peristaltic efficiency and limiting the active secretion of water, sodium, and chloride ions by the intestinal epithelial cells [48] as well as due to its anti-inflammatory activity [49]. Of note, the depletion of SCFA and SCFA-producing organisms has been observed in PD patients [29] and in other synucleinopathy conditions such as multiple system atrophy [50]. Supporting this, the enhanced inflammation, as a consequence of alterations in the immune response to both commensal and harmful bacteria, has been suggested to induce LP, since a significant association between some polymorphisms in peptidoglycan recognition protein-encoding genes and an increased risk of developing PD has been demonstrated [51].

Another analysis of microbiota composition in PD pointed to differences in microbiota metabolism, namely in β-glucuronate and tryptophan degrading pathways [36,37]. This is in line with previous research findings demonstrating an increased level of tryptophan metabolites including kynurenine (KYN), xanthurenic acid, and hydroxyanthranilic acid in the urine of idiopathic PD patients, compared with that of controls [52]. The increased tryptophan metabolism is of particular interest, as it is a precursor of serotonin and is decreased in PD patients’ brains. In addition, tryptophan catabolites, notably KYN, have been reported to disrupt epithelial barrier integrity and induce inflammation in the intestine as well as could be a source of systemic neuropathology [53]. Keshavarzian et al. [31] have demonstrated a reduction in normal metabolic pathways and an increase of metabolic activities devoted to lipopolysaccharide biosynthesis and type III bacterial secretion systems. Supporting this, Tetz et al. [38] based on a detailed comparative metagenomic analysis of intestinal phagobiota in PD patients and non-parkinsonian individuals, have identified shifts of the phage/bacteria ratio in lactic acid bacteria, producing dopamine and regulating intestinal permeability, which are major factors implicated in PD pathogenesis. These findings seem to support the concept of gut-originating and inflammation-driven PD pathogenesis. Interestingly, Forsyth and co-workers [30] have demonstrated that the changes in bacterial population composition and increased intestinal permeability positively correlated with the increased level of intestinal α–synuclein in PD patients. The positive feedback loop involving the gut inflammation in conjunction with the expression of α-synuclein in the enteric neurites of the upper GI tract has also been demonstrated in pediatric patients, who suffer from acute and chronic inflammation in the intestinal wall [54].

Another intestinal feature of PD widely reported is the presence of enteric abnormalities in α-synuclein, which is expressed as a normal component of the ENS [55]. Numerous pathological studies indicate, however, that the occurrence of α-synuclein in the intestine of PD patients is more expressed than in age-matched healthy controls [30,56,57,58,59,60]. Of note, the increased α-synuclein immunoreactivity has also been demonstrated in intestinal biopsies collected from clinically healthy individuals, who later experienced an onset of PD [57,59]. It is reasonable to speculate that α-synuclein pathology is present before CNS neurodegeneration, as an over-expression of α-synuclein correlates positively with the prevalence of its aggregations in both the intestines and brains. In line with this, α-synuclein inclusions have been almost consistently shown to occur in the GI tract of PD patients in all stages of the disorder [61,62,63]. Phosphorylated and aggregated α-synuclein has been detected in the esophagus, stomach, small intestine, colon, and rectum of PD patients [64,65,66,67,68]. Some authors have also reported the presence of LBs and/or LNs in GI tissue samples [7,46,55,62]. The accumulation of LP has been demonstrated to be present in the peripheral autonomic nervous system (PANS), including the ENS and autonomic ganglia [7,55,69,70]. There are studies showing the presence of a rostrocaudal gradient of α-synuclein within the GI tract [65,70] with the highest frequency of prevalence in the submandibular gland and lower esophagus, followed by the stomach, small bowel, colon and rectum [71]. Of note, this gradient coincides with the distribution of vagal innervation from the DMV [71], which has been shown to be also affected in PD [69,72,73,74]. In addition, the involvement of spinal and peripheral sympathetic and parasympathetic neurons by α-synuclein pathology has been reported [65,69,75].

The involvement of the synucleinopathy of a central or peripheral autonomic system likely reflects the pathophysiology of gastrointestinal symptoms in PD patients since it controls motility and secretion in GI. Constipation and impaired defecatory function may reflect α-synuclein pathology in myenteric and submucosal neurons, which were almost constantly affected in PD [56,61,62,64,70,76]. Lebouvier et al. [61] have reported a correlation between the burden of α-synuclein inclusions in the submucosal plexus in biopsies of the ascending colon and the presence of chronic constipation [61]. Upper gastrointestinal motility is primarily controlled by the DMV. Therefore, esophageal and gastric dysmotility may be associated with affected by α-synuclein pathology DMV, which was shown in PD patients [69,72,73,74]. Several studies showing the presence of α-synuclein inclusions in salivary glands and the submandibular gland [65,67,77,78] have suggested its involvement in the impairment of salivary function in PD. In addition, the presence of LP in the neurons of the olfactory system of PD patients has been reported [74,79,80] to be likely involved in the loss of smell observed in the early stage of the disease [81].

Pathological investigations have revealed a specific chronological and regional pattern during the progression of PD, on the basis of which Braak and colleagues developed a pathological staging system [5,82,83]. At this time, the pathogenesis of PD remains poorly understood. Nonetheless, there is considerable evidence that the olfactory bulb, the ENS, the intermediolateral nucleus of the spinal cord and the dorsal motor nucleus of the vagus are the sites of early involvement [69,75,82,84]. This concept also presents a potential explanation as to why the majority of PD patients exhibit the early and severely affected DMV and the olfactory bulb [85]. Therefore, the ‘dual-hit hypothesis’ postulates that α-synuclein pathology might initially be triggered by exogenous insults targeting the gut and olfactory system. Its anterograde progress from the olfactory system into the temporal lobe, and the retrograde transport to the brainstem from the gut, seems to likely reflect the observed symptoms in a subgroup of PD patients with young-onset and long duration of disease [86]. This scenario reflects the direct contact between environmental agents and neurons of the olfactory bulb and ENS through inhalation or ingestion, respectively, and the prion-like transfer of the abnormal α-synuclein along preganglionic parasympathetic fibres to the DMV. Supporting this, Gray and collaborators [87], who have revealed the relative abundance of α-synuclein within macrophages in the vermiform appendix in patients with no history of neurological disease, suggested this anatomical locus as a candidate for the initiation of the “prion-like” cascade in PD pathogenesis.

The concept that the vagal nerve constitutes a major highway for spreading of α-synuclein pathology into the CNS is supported by epidemiological studies on a large scale, showing that vagotomy could be protective against PD. Two independent studies have reported that the risk of PD may be substantially decreased following truncal vagotomy [28,88]. Finally, some evidence supports the scenario that synucleinopathy spreads in prion-like fashion into the brain in a human subject. This concept presents a potential explanation as to why Lewy bodies were present, at autopsy, in the grafted neurons of fetal midbrain tissue in the striatum of Parkinson’s disease patients 1–2 decades after surgery [11,12,13,89].

## 4. Evidence from Experimental Models

Different lines of approach in animal model systems have been used to examine the role of the brain-gut-(microbiota) axis and the idea of prion-like spreading of α-synuclein in the context of PD. These include the use of pesticide-induced synucleinopathy or xenograft models, germ-free or exposed to antibiotics animals, transgenic, engineered by viral vector or wild types animals as well as the use of different surgical procedures (Table 2).

In view of Braak’s theory, an initial α-synuclein pathology might be triggered by exogenous insults targeting the gut and/or olfactory system. Besides stochastic events leading to the de novo aggregation of endogenous α-synuclein, exogenous agents such as toxins or inflammatory agents are proposed to initiate the pathology [90].

Since environmental exposure to pesticides has been reported to be associated with an increased risk of PD and the high expression of α-synuclein in the brain [91], intragastric administration of the pesticide rotenone to rodents has been used to promote misfolding of α-synuclein in the ENS. Pan-Montojo and co-workers [25] have demonstrated that α-synuclein appears in the DMV, intermediolateral nucleus of the spinal cord and the SNpc in mice treated *i.g.* with rotenone [25]. It was later shown that the rotenone’s effect on PD-like disease progression might be prevented by hemivagotomy or the resection of autonomic nerves [26]. The Braak’s theory concerning the retrograde transport of the α-synuclein pathology from the ENS to the CNS was verified by Phillips et al. [92], who have examined the pattern of expression of the protein in the autonomic circuitry of the proximal gut in aged rats. They have demonstrated the presence of α-synuclein in vagal efferent axons and terminals originated in the DMV and in addition some of these preganglionic efferents terminated on α-syn-positive neurons in the myenteric plexus of the stomach and duodenum. Vagotomy eliminated α-synuclein distribution to most neurites in the plexus [92]. Also, findings of Noorian et al. [27] in transgenic mice have supported the idea that vagal innervation may be a critical factor for the development of α-synuclein neuritic pathology in the GI tract since vagotomy resulted in the complete elimination of α-synuclein in the appropriate terminal distribution. Remarkably, Ulusoy and co-workers [93] have postulated the ability of α-synuclein to move both anterogradely and retrogradely as they demonstrated that the protein is also transferred from the brain to peripheral tissues via the DMV. Of note, similar to clinical evidence, gastrointestinal problems such as declined gastrointestinal motility, has been observed in transgenic mice expressing mutant α-synuclein aggregates in the ENS ganglia [27,94], which appeared before motor dysfunctions [94]. There is also experimental evidence supporting Braak’s hypothesis concerning the α-synucleinopathy in the olfactory bulb. In Tg mice overexpressing A53T-mutant human α-synuclein, the protein appeared in the olfactory bulb at an earlier stage than in the substantia nigra and its distribution positively correlated with age [95].

As was shown in the previous section, the changes in the composition of the gut microbiota correlate well with PD symptoms which, on the other hand, are associated with more extensive α-synuclein deposition within the enteric nervous system. However, until now, it has not been clear if the changed gut microbiota plays a causative role in the pathogenesis of PD or is simply a consequence of the disease [96,97]. The possibility of the pathophysiological influence of gut bacteria on the neuro-immune system, in intestinal dysfunctions and CNS neurodegeneration, has been examined in several animal models of PD. Recently, Yang et al. [98] have demonstrated that in mice with rotenone-induced PD microbiome dysbiosis, colonic inflammation, enteric abnormal α-synuclein accumulation, and gastrointestinal dysfunction appeared before motor dysfunction and CNS pathology. The gut dysbiosis was characterized by an overall decrease in bacterial diversity and an increase in Firmicutes/Bacteroidetes ratio and consequent changes in 11 metabolic pathways. This finding supports the view that alterations of the enteric bacteria-immune network could contribute to CNS pathology [98]. Gut dysbiosis and/or small intestinal overgrowth have been suggested to induce over-response of the immune system and consequently systemic and/or CNS inflammation [32,47]. Sampson and co-workers [99] have demonstrated that α-synuclein in transgenic mice under germ-free (GF) conditions displayed reduced abnormal α-synuclein accumulation in the brain. Additionally, a positive feedback loop involving decreased GI and motor dysfunctions and microglia activation in conjunction with GF conditions and bacterial depletion by antibiotics has been demonstrated in animals overexpressing α-synuclein. Based on these observations it could be suggested that gut microbiota contributes to central PD pathology by potentiating microglial activation and neuroinflammation and related motor and GI dysfunctions and, importantly, that treatment with antibiotics can provide an improvement of gastrointestinal and central symptoms of the disease. Moreover, microbiota transplants from PD patients enhanced gut dysfunctions and motor impairment as compared to microbiota transplantation from healthy controls [99]. In aged Fischer rats, treated with *E. coli* producing bacterial amyloid protein curli, which is a β-sheet-rich protein capable of cross-seeding, an increased neuronal expression of α-synuclein has been demonstrated both in the submucosal and myenteric plexus as well as in the brain, which correlated well with enhanced microgliosis and astrogliosis [100]. The relation between alterations of the intestinal epithelial barrier and enteric inflammatory responses was investigated in animals with experimental PD induced by lipopolysaccharide (LPS) [101]. In this study, an abnormal intestinal permeability appeared in the early phase of the disease, before α-synuclein accumulation in the ENS and SN [101]. Moreover, a recent study has demonstrated an increased expression of α-synuclein in the cortex of peptidoglycan recognition protein 2 (Pglyrp2)-knockout mice, supporting the idea that disrupted gut flora and immune response, as seen in the Pglyrp2-knockout animal could be a causative factor in PD [102]. Taken together, these studies highlight the crucial roles of dysbiosis in the initiation and/or progression of the neurodegeneration, often associated with activation of neuroinflammatory processes. In this concept, an abnormal microbiota composition (or anomalous host response to a normal microbiota) leads to a local immune response as well as an altered level of bacterial metabolites. These may evoke impairment in the gastrointestinal mucosal barrier (“leaky gut”) that enables migration of gut bacteria and inflammatory cytokines, triggering a systemic inflammatory response, which, in turn, impairs the blood-brain barrier and promotes neuro-inflammation and ultimately, neuronal degeneration. These effects may be exacerbated by abnormal signaling through the vagus nerve, caused by some microbial metabolites acting as neurotransmitters and neuromodulators [103,104].

Considerable efforts have been undertaken to study, both within the ENS and CNS, spreading routes with a focus on understanding the plausibility of the prion-like behavior of α-synuclein pathology. Since currently, it is not possible to monitor precisely progress of the LP in an individual, different animal models have been developed to trigger and subsequently examine synucleinopathy at different time points. Injections of rodents with exogenous α-synuclein have been demonstrated to initiate a cascading seeding event from the injection site that has been interpreted as a prion-like behavior of α-synuclein pathology. Preformed fibrillar α-synuclein assemblies (PFFs) injected intraperitoneally or intraglossally led to the distribution of α-synuclein pathology to the brain and spinal cord [105]. α-Synuclein from the human PD brain injected into the stomach [106] or intestine [107] of rodents is transferred to the myenteric neurons [106] as well as to the DMV via the vagal nerve in a time-dependent manner [107]. After a single peripheral intramuscular injection of recombinant fibrillar α-synuclein, its pathological inclusions were also detected in the spinal cord, brainstem, midbrain, and cortex [108]. When PFFs were injected into the olfactory bulb, they triggered the spread of aggregated α-synuclein pathology in an anatomical pattern within the brain [109]. Intracerebral inoculations with preformed recombinant human or mouse fibrils of α-synuclein have been shown to promote the formation and spread of α-synuclein pathology, including LB/LN-like inclusions, in the brain [110,111,112,113,114,115] and even along the other part of the CNS [116,117]. The acceleration of α-synuclein aggregation in the brain of presymptomatic animals also followed intracerebral inoculation of brain tissue from mice or patients affected by the synucleinopathy [112,116,117,118,119,120]. A delivery of synuclein with Lewy-like pathology into the brain with viral vectors led to the same effects [93,110,113,121]. In addition, Angot et al. [122] have reported that hα-syn transferred from a host brain, with engineered nigral neurons, to grafted embryonic ventral mesencephalon (VM). Similarly, transmission of hα-syn from host to grafted mouse cortical neural stem cells was observed in transgenic mice expressing human α-synuclein [123]. Chandra et al. [124] have discovered the presence of hα-syn in enteroendocrine cells (EECs) in transgenic mice expressing human α-synuclein and endogenous green fluorescent protein in cholecystokinin cells. Consistently with this massive burden of α-synuclein pathology, early onset of neurological symptoms have been revealed in conditions including: a reduction in DAergic neurons [113,114,115,116,120], induction of cell death [123], loss of neurotransmitters such as dopamine [115] and enkephalin [112], inflammatory response [106,108,110,111,118], impairment of synaptic physiology [113] and protein degradation [105,111]. Consequently, the acceleration and increased accumulation of α-synuclein pathology was associated with motor [94,105,108,111,113,115,117,118,119,120] or olfactory impairment [109] and a reduction in survival rate [108,116,118]. These findings seem to be convincing and indicate that α-synuclein assemblies can spread. Moreover, several studies in animal models have provided insight into mechanisms underlying this process. Angot et al. [122] have shown that in rats driven by AAV2/6 vector α-synuclein aggregation, α-synuclein is transferred to grafted dopaminergic neurons in the not aggregated and not phosphorylated form with the involvement of endocytosis for α-synuclein uptake. This possibility was raised recently by Mao et al. [115] in TgLag3 mice, who have confirmed that the cell-to-cell transmission of misfolded α-synuclein involves the uptake of the pathogenic particle from the outside of the cell by endocytosis, that is mediated by the cell surface receptor LAG3 or neurexin 1β and amyloid β precursor-like. Finally, the recent discovery in transgenic mice by Chandra et al. [124] demonstrated that sensory cells of the gut EECs, which are exposed to pathogen or toxin in the gut lumen, contain α-synuclein. Since the functional synaptic connection between EECs and enteric nerves was established, it may provide a possible explanation for how PD may originate in the gut. Collectively, all the above-discussed data from animal studies seem to support the concept of the gut-to-brain transmission of PD pathology in the “prion-like’’ fashion.

## 5. Does Parkinson’s Disease Actually Originate in the Gut?

All the above-summarized data seem to be convincing and supporting the concept of a gut-to-brain transmission of PD pathology. However, when reviewing the available literature, we also came across a doubt as to whether these data actually describes the disease’s development in all PD patients. Of note, there are a few reports indicating that not all PD cases strictly follow Braak’s staging system. Kalaitzakis et al. [125] reported that 47% of PD cases coming from a UK tissue bank did not match the predicted caudorostral distribution of LP in the brain, including the absence of prominent DMV pathology in 7% of cases. Similarly, the Braak’s scheme did not fit in 18.3% of PD cases from the Vienna tissue brain bank, including 8.3% in which the DMV was not involved at all [126]. Moreover, the hypothesis of peripheral onset of PD is questioned, since there is the relative lack of postmortem cases with a presence of α-synuclein pathology in the PNAS without concomitant CNS involvement. Only Fumimura et al. [127] have demonstrated the occurrence of LBs in the adrenal glands, but in only 2 cases out of 783, in which the pathology was not detected in the CNS. Also, a protective effect of truncal vagotomy demonstrated by Svensson et al. [28] has been questioned by a Norwegian research group [128], who independently reanalyzed the Danish vagotomy dataset. As Tysnes and colleagues [128] included only 4050 patients with full truncal vagotomy, in contrast to 5339 patients from the study by Svensson et al. [28], any significant difference for the risk of PD between the vagotomy group and reference matched populations has been shown [128]. Supporting this, recently, Recasens et al. [129] have argued against a pathogenic ability of peripheral α-synuclein assemblies in promoting α-synuclein pathology in the brain. In their study intracerebral inoculation of mice with α-synuclein aggregates from postmortem PD stellate ganglia (paravertebral sympathetic ganglion) triggered neither nigrostriatal neurodegeneration nor α-synuclein pathology in the SNpc [129]. However, contrary to these findings in their previous report nigral LB extracts from PD patients administered intracerebrally to animals induced α-synuclein pathology, neuroinflammation and finally neurodegeneration in the SNpc [120].

## 6. Concluding Remarks

Based on the gathered evidence from human and animal research that has been published over the last few decades, it is likely that α-synuclein pathology originating within the gastrointestinal tract spreads via a prion-like mechanism. As depicted in Figure 1 there is growing concern within the scientific communities about this concept in PD pathology.

Given the abundance of evidence, it could be speculated that in PD, the gut is affected at the earliest. PD patients are characterized by abnormal gut microbiota composition, impairment of the intestinal barrier and enteric neuro-immune system that lead to enteric inflammation that contributes to neuroinflammation and neurodegeneration in the CNS. This data has been supported by findings from animal experiments. Other lines of evidence indicate that the intestine is the site where the aggregation of endogenous α-synuclein occurs initiating the progression of PD. It has been widely supported by reports, demonstrating that Lewy pathology appears in peripheral tissues up to 20 years prior to PD diagnosis. There is considerable evidence that the olfactory regions might also be the starting point of synucleinopathy which can be explained by the fact that their neurons are exposed directly to environmental agents. In line with this, constipation and hyposmia appear years or decades prior to PD diagnosis. So far, the hypothesis that LP propagates from the ENS by trans-synaptic cell-to-cell transmission through sympathetic and parasympathetic nerves to the DMV into the CNS is controversial. As some PD patients do not follow Braak’s staging system [125,126], there is criticism concerning the pattern of spreading of α-synuclein pathology [47,130]. On the other hand, lack of standardized methods of α-synuclein detection and the inability to detect all its pathological forms are suggested to be the reason for differences in the experimental findings [85,130]. Nevertheless, studies reporting the decreased risk of PD following truncal vagotomy support the route of α-synuclein pathology from the ENS to the CNS. The different findings of Norwegian and Danish studies could be explained by not imposing, in the Norwegian analysis, a minimum follow-up time from vagotomy to PD diagnosis required for exclusion patients with the conceivable pathology in the brain stem already at the time of vagotomy [85]. The convincing evidence of the prion-like mechanism of α-synuclein pathogenesis came from postmortem observations that pathological α-synuclein inclusions appeared in healthy grafted neurons. Finally, accumulating evidence from animal research demonstrates spreading of α-synuclein pathology following peripheral administration of its pathological aggregates. However, in the light of the recent report of Recasens et al. [129] further studies are required to identify the exact composition and structure of LP from the PNAS and CNS in order to provide insight into a possible maturation of α-synuclein needed for acquiring pathogenic features. In the context of the “prion-like” mechanism of PD, it should be noted that a few experimental studies have reported endocytic mechanisms involving contact of α-synuclein with LAG3 receptors. Since the receptors are expressed on immune cells and/or neurons of the GI tract, it supports the hypothesis that PD may indeed originate within the gastrointestinal tract.

In this review, we frame current evidence for the gut-to-brain hypothesis of PD in the context of prion-like spreading of α-synuclein. However, much remains to be discovered regarding the gut bacteria-neuro-immune crosstalk, the route for the spread of α-synuclein pathology from the ENS to the CNS and its cellular transmission. It is believed that standardization of α-synuclein detection methods and the development of techniques that enable the observation of its dynamic tracking, including transport within cells, will provide tools for elucidation of presented hypotheses [85,130].

## Figures and Tables

**Table 1:** Summary of evidence from research in human subjects for concepts of the gut-brain axis and the prion-like spreading/propagation of α-synuclein in Parkinson’s disease.

Subjects/Specimens	Outcome/Results	Reference
**Gut Dysbiosis/SIBO**		
Intestinal biopsy samples from PD subjects and HC	↑ intestinal permeability (↑ urinary sucralose excretion, ↓ plasma LBP level) ↑ *E. coli* in lamina propria and epithelium ↑ inflammation/oxidative stress (↑ 3-NT) ↑ α-syn immunoreactivity in PD patients	Forsyth et al. 2011 [30]
Faecal samples from PD patients and cohabitants	↓ total fecal bacterial ↓ *Clostridium coccoides* (*C. leptum*) and *Bacteroides fragilis* ↑ *Lactobacillus* *L. gasseri* counts correlated positively with disease durations and negatively with stool frequencies *C. coccoides* counts correlated negatively with disease durations and positively with stool frequencies ↑ intestinal permeability (↓ serum LBP level) in PD patients	Hasegawa et al. 2015 [33]
Sigmoid mucosal biopsy and faecal samples from PD subjects and HC	↑ putative proinflammatory bacteria from the family Oxalobacteraceae (Proteobacteria, class Betaproteobacteria) and genus *Ralstonia* (Proteobacteria, class Betaproteobacteria, family Oxalobacteraceae) in the mucosa ↓ putative “anti-inflammatory” butyrate-producing bacteria from the genera *Blautia*, *Coprococcus*, and *Roseburia* in faces ↓ microbiome genes involved in normal metabolic activities of microbiota ↑ microbiome genes involved in bacterial secretion, LPS, and ubiquinone biosynthesis in PD patients	Keshavarzian et al. 2015 [31]
Fecal samples from PD patients and HC	↓ Prevotellaceae ↑ Enterobacteriaceae positively correlated with the severity of postural instability and gait difficulty ↑ Verrucomicrobiaceae and *Bradyrhizobiaceae* associated with the degree of constipation in PD patients	Scheperjans et al. 2015 [34]
Fecal samples from PD patients and HC	↓ *Dorea, Bacteroides*, *Prevotella*, *Faecalibacterium, Bacteroides massiliensis*, *Stoquefichus massiliensis*, *Bacteroides coprocola*, *Blautia glucerasea*, *Dorea longicatena*, *Bacteroides dorei*, *Bacteroides plebeus*, *Prevotella copri*, *Coprococcus eutactus*, and *Ruminococcus callidus* ↑ *Christensenella*, *Catabacter*, *Lactobacillus*, *Oscillospira, Bifidobacterium*, *Christensenella minuta*, *Catabacter hongkongensis*, *Lactobacillus mucosae*, *Ruminococcus bromii*, and *Papillibacter cinnamivorans* in PD patients	Petrov et al. 2016 [35]
Fecal samples from PD patients and HC	↓ phyla Bacteroidetes and Prevotellaceae ↓ *Lactobacillaceae* and *Enterococcaceae* ↓ *Faecalibacterium prausnitzii* ↑ *Bifidobacterium* and *Enterobacteriaceae* ↓ SCFAs (acetate, propionate and butyrate) in PD patients	Unger et al. 2016 [29]
Stool samples from PD patients and HC	↑ Verrucomicrobiaceae (genus *Akkermansia*, including *Akkermansia muciniphila* and *Alistipes shahii.*) ↑ Firmicutes ↓ Prevotellaceae (genus *Prevotella*, including *Prevotella copri*) ↓ Erysipelotrichaceae (genus *Eubacterium*, including *Eubacterium biforme*) ↓ *Clostridium saccharolyticum* ↓ microbiome glucuronate pathway (d-Glucuronate degradation, d-glucuronate → pyruvate, and d-glyceraldehyde) ↑ microbiome tryptophan pathway (tryptophan→kynurenine→2-aminomuconate) in PD patients	Bedarf et al. 2017 [36]
Stool samples of PD patients and HC	↑ *Bifidobacteriaceae*, *Christensenellaceae*, *Tissierellaceae*, *Lactobacillaceae*, and *Verrucomicrobiaceae families* ↓ *Lachnospiraceae*, *Pasteurellaceae* 26 microbiome’ metabolic pathways changed in PD patients	Hill-Burns et al. 2017 [37]
Faecal samples from drug-naive PD patients HCs.	↓ *Prevotellaceae*, *Lachnospiraceae, Lactobacillaceae* and *Streptococcaceae* ↓ phages belonged to the *Siphoviridae* family (*Bacillus*, *Enterobacteria, Lactococcus Streptococcus*, and *Salmonella* phages) and *Lactobacillus* phages of the *Myoviridae* family ↑ phages belonged to the *Siphoviridae* family (*Leuconostoc, Lactococcus*, and *Enterobacteria* phages) *Enterobacteria* phages of the *Myoviridae* family, and *Salmonella* phages of the *Podoviridae* family ↑ phage/bacteria ratio for *Lactococcus* in PD patients	Tetz et al. 2018 [38]
breath samples from PD patients and HC	SIBO (↑ hydrogen level in GBT) ↑ bloating and flatulence ↑ motor complications (UPDRS-III) in PD patients	Gabrielli et al. 2011 [39]
Breath samples from PD patients and HC	SIBO (↑ hydrogen and methane levels in GBT and LBT) ↑ motor complications (UPDRS-IV) in PD patients	Fasano et al. 2013 [40]
Breath samples from PD patients and HC	SIBO (↑ hydrogen in LBT) in early stage PD	Tan et al. 2014 [41]
Urinary samples from PD patients and HCs	SIBO (↑ urine indican concentrations) in PD patients	Cassani et al. 2015 [42]
**Peripheral distribution of α-synuclein pathology**		
Autopsy GI tissue samples from PD subjects	LBs in the myenteric plexus of the lower oesophagus	Wakabayashi et al. 1988 [7]
Postmortem study of the brain from PD subjects	LBs in TH-positive neurons in the midbrain, pons, medulla (DMV), rostral medial lemniscus, raphe obscurus nucleus, dorsal tegmental bundle	Halliday et al. 1990 [72]
Autopsy GI specimens from PD subjects	LBs in the paravertebral and celiac sympathetic ganglia and in the Auerbach’s plexus of the lower upper, middle and lower esophagus, duodenum, ileum, descending colon and rectum, and Meissner’s plexuses of the duodenum and descending colon	Wakabayashi et al. 1990 [70]
Postmortem study of the brain from PD subjects	LBs in DMV	Wakabayashi et al. 1999 [73]
Postmortem study of the brain from PD subjects	LNs and LBs in non-catecholaminergic neurons of the dorsal glossopharyngeus-vagus complex, projection neurons of the intermediate reticular zone, olfactory bulb, olfactory tract, and/or anterior olfactory nucleus	Del Tredici et al. 2002 [74]
Autopsy specimens of brains, spinal cords and PANS from PD subjects	LNs in the intermediolateral nucleus of the thoracic spinal cord, sacral parasympathetic cell column of the sacral spinal cord, dorsal and anterior horns, paravertebral chain ganglia, vagus nerve and DMV	Bloch et al. 2006 [69]
Autopsy stomach specimens from PD subjects	α-syn aggregations, including LNs and LBs in the gastric wall (peripheral nerve, fibers generated from the Auerbach plexus, nerve fiber bundle of Meissner’s plexus), gastric Meissner plexus and Auerbach plexus	Braak et al. 2006 [55]
Postmortem study of the brain and complete spinal cords from PD subjects	α-syn aggregations, including LBs and LNs, in spinal cord lamina I neurons, parasympathetic preganglionic projection neurons of the vagal nerve, sympathetic preganglionic neurons of the spinal cord, postganglionic neurons of the coeliac ganglion	Braak et al. 2007 [75]
Autopsy brain tissues from PD subjects	LBs and LNs in the present in olfactory bulb and tract, anterior olfactory nucleus, orbitofrontal cortex, amygdala and hippocampus	Hubbard et al. 2007 [79]
Colonic biopsies from PD patients	p-α-syn immunoreactivity in the submucosal neurites	Lebouvier et al. 2008 [64]
Olfactory bulb sections from PD patients	synucleinopathy in the olfactory bulb olfactory bulb synucleinopathy density scores correlated significantly with: those in other brain regions (anterior medulla, anterior pons, midbrain, amygdala, gyrus, inferior parietal lobule) mini mental state examinations and UPDRS scale (motor part) score	Beach et al. 2009 [80]
A whole-body autopsy of PD subjects	p-α-syn immunoreactivity in the spinal cord and vagus nerve as well as in the submandibular gland, in the submucosa of the lower esophagus, in the stroma of the pancreas, in the submucosa of a primary bronchus, in the submucosa of the larynx, in the adrenal medulla, in the stroma of the parathyroid gland, and in the ovary	Beach et al. 2010 [65]
Colonic biopsies from PD patients	p-α-syn immunoreactivity in the submucosal neurites and submucosal plexus LP positively correlated with dysarthria and postural instability, constipation severity	Lebouvier et al. 2010 [61]
Gastrointestinal specimens from patients with no history of neurological and psychiatric diseases	α-syn mRNA expression in full-thickness sections and intestinal wall layers (submucosa and tunica muscularis) α-syn mRNA expression in myenteric ganglia. α-syn immunoreactivity in ganglia of the myenteric and submucosal plexus as well as in intramuscular nerve fibers p-α-syn immunoreactivity in ganglia of the myenteric and submucosal plexus	Böttner et al. 2012 [76]
Colonic biopsies from PD patients	p-α-syn immunoreactivity (including LNs) in the ascending colon, descending colon and rectum	Pouclet et al. 2012 [62]
Colonic biopsies from PD patients	α-syn within neuronal tissues in the colonic submucosa	Shannon et al. 2012 [56]
Colonic biopsies from PD patients	α-syn-positive structures in colonic submucosa 2 to 5 years before the first reported symptom of PD	Shannon et al. 2012 [57]
Submandibular gland autopsy in an elderly subject with PD	p-α-syn immunoreactivity in submandibular gland	Beach et al. 2013 [78]
Colonic specimens from PD patients	α-syn expression in myenteric and submucosal ganglia and extraganglionic nerve fibers	Gold et al. 2013 [58]
Submandibular gland biopsies from PD patients	p-α-syn immunoreactivity in nerve fibers or puncta within submandibular salivary gland tissue	Adler et al. 2014 [77]
Multiorgan (including GI tract) postmortem study of PD subjects	p-α-syn/α-syn immunoreactivity in the distal esophagus, stomach, ileum, colon, and rectum (ganglia of the myenteric plexus)	Gelpi et al. 2014 [66]
GI tissue samples from patients with no history of neurological disease	α-syn immunoreactivity in the mucosal plexus of the vermiform appendix α-syn immunoreactivity in the CD68-immunoreactive macrophages in the appendiceal lamina propria α-syn immunoreactivity in the mucosa of the stomach, ileum and colon	Gray et al. 2014 [87]
GI tissue samples from PD patients	p-α-syn immunoreactivity in the stomach, small intestine, large intestine, α-syn-positive neurites within the lamina propria and submucosal myenteric nerve fibres α-syn small rounded inclusions within submucosal ganglia α-syn-positive biopsies correlated with autonomic symptoms (sometimes prior to motor symptoms)	Hilton et al. 2014 [59]
Colonic biopsies from PD patients	p-α-syn immunoreactivity (LBs) in the submucosa ↓ occludin expression	Clairembault et al. 2015 [46]
Stomach biopsies from PD patients	α-syn immunoreactivity (antrum, pylorus, and duodenum)	Sánchez-Ferro et al. 2015 [68]
Colonic mucosal biopsies from PD patients	α-syn and p-α-syn immunoreactivity in individuals with early PD and with later PD > control subjects	Visanji et al. 2015 [60]
colonic biopsies from PD patients	LTS in the lamina propria of the mucosa, submucosa, and epithelium	Corbillé et al. 2016 [63]
PD Multiorgan (including GI tract) postmortem study of PD subjects	p-α-syn/α-syn immunoreactivity in the nasal region, oral region, salivary gland, esophagus, stomach, small intestine and appendix (mucosa, submucosal ganglia, intramuscular nerve fibers, and myenteric ganglia)	Stokholm et al. 2016 [67]
**Vagotomy and risk of PD**		
A cohort of individuals underwent vagotomy and matched general population (control)	↓ risk of PD in patients who underwent full truncal vagotomy	Svensson et al. 2015 [28]
Vagotomized patients (truncal, selective, and unknown); reference individuals	↓ risk of PD in patients with truncal vagotomy vs. selective vagotomy ↓ risk of PD in patients with truncal vagotomy vs. reference individuals (ns)	Liu et al. 2017 [88]
**Prion-like spread of α-synuclein**		
An individual with PD transplanted bilaterally with human ventral mesencephalon derived from embryos—a post-mortem examination	↑ α-syn and ubiquitin immunoreactivity ↓ DAT immunoreactivity in grafted neurons 14 years after transplantation	Kordower et al. 2008 [11]
An individual with PD transplanted bilaterally with human ventral mesencephalon derived from embryos—a post-mortem examination	↑ α-syn, ubiquitin and thioflavin S immunoreactivity ↓ DAT an TH immunoreactivity in grafted neurons 14 years after transplantation	Kordower et al. 2008 [12]
PD subjects transplanted fetal mesencephalic dopaminergic neurons—a post-mortem examination	↑ α-syn immunoreactivity ↓ TH-positive neurons in grafted neurons 11-16 years after transplantation	Li et al. 2008 [13]
PD subjects transplanted fetal mesencephalic dopaminergic neurons—a post-mortem examination	↑ α-syn immunoreactivity ↓ DAT immunoreactivity ↓ TH-positive neurons in grafted neurons 13–22 years after transplantation	Kurowska et al. 2011 [89]

↑ = increase, ↓ = decrease; 3-NT—3-nitrotyrosine; α-syn—α-synuclein; DAT—dopamine transporter; DMV—vagal dorsal motor; GBT—glucose breath test; HC—healthy control; LBP—lipopolysaccharide-binding protein; LBs—Lewy bodies; LBT—lactulose breath test; LNs—Lewy neurites; LP—Lewy pathology; LTS—Lewy type synucleinopathy; LPS—Lipopolysaccharide; PANS—peripheral autonomic nervous system; p-α-syn—phosphorylated α-synuclein; PD—Parkinson’s disease; SCFAs—short-chain fatty acids; SIBO—small intestinal bacterial overgrowth; TH—tyrosine hydroxylase; UPDRS—Unified PD Rating System.

**Table 2:** Summary of evidence from animal studies for concepts of the gut-brain axis and prion-like propagation of α-synuclein in Parkinson’s disease.

Experimental Model	Outcome/Results	Reference
**Dysbiosis**		
ASO mice under GF condition	↓ p-α-syn and aggregation-specific α-syn immunoreactivity in the CP and SN ↓ α-syn insoluble expression ↑ numbers and total lengths of microglia branches within the CP and SN (↓ activation state) ↓ Iba-1 immunoreactivity in microglia in the CP ↓ TNF-α and IL-6 in the CP, Mid and FC ↑ motor function (beam traversal, pole descent, and hindlimb clasping reflexes) ↑ fecal pellets ↓ SCFAs production	Sampson et al. 2016 [99]
ASO mice under SPF condition	↑ motor function (beam traversal, pole descent, and hindlimb clasping reflexes) ↓ Iba-1 immunoreactivity in microglia in the CP ↓ microglia diameter in the CP and SN (↓ activation state) ↑ fecal pellets ↓ SCFAs production	Sampson et al. 2016 [99]
ASO mice treated (*p.o.*) with microbiota from PD patients	↓ fecal pellets ↓ motor function (beam traversal, pole descent and nasal adhesive removal)	Sampson et al. 2016 [99]
Male C57BL/6 mice treated with rotenone (*i.g.*)	↓ Bacteroidetes ↑ Firmicutes and Firmicutes/Bacteroidetes ratio ↑ 11 metabolic pathways ↑ α-syn and p-α-syn immunoreactivity in the colon ↑ α-syn and p-α-syn immunoreactivity in the midbrain ↓ colon motility and stool water content ↓ locomotor deficits in the open field and pole tests ↑ TNF-α, IL-6 and iNOS expression ↑ TLR2 expression	Yang et al. 2017 [98]
**Peripheral distribution of α-synuclein**		
Subdiaphragmatic vagotomy in aged Fischer 344 and SD rats	↓ α-syn-positive varicosities within ganglia and α-syn-positive axons in fiber bundles	Phillips et al. 2008 [92]
Tg A53T and A30P mice	α-syn immunoreactivity within myenteric and submucosal plexuses ↓ WGTT and colonic motility ↓ motor behavior (open field test, rotarod test)	Kuo et al. 2010 [94]
C57BL/6J mice treated with rotenone (*i.g.*)	↑ α-syn phosphorylation, accumulation and aggregation with gliosis in ENS ganglia ↑ intracellular and axonal α-syn in the spinal cord and the brainstem (IML and DMV); the α-syn pathology progressed into the SN	Pan-Montojo et al. 2010 [25]
TgA53T mice	α-syn immunoreactivity in the olfactory bulb (glomerular, mitral, and granule layers) high α-syn immunoreactivity in the calcium binding protein-positive cells low α-syn immunoreactivity in the Nissl-positive cells	Ubeda-Bañon et al. 2010 [95]
TGA53T mice injected with human brain tissue extract containing LBs into the stomach wall	↑ α-syn deposition in the myenteric neurons ↑ activation of macrophages (MHC-II expression)	Lee et al. 2011 [106]
Subdiaphragmatic vagotomy in TgA53T mice	↓ α-syn immunoreactivity in the myenteric plexus of the dorsal and ventral stomach and duodenum	Noorian et al. 2012 [27]
Hemivagotomy or partial sympathectomy of the nervus mesentericus inferior in mice orally treated with rotenone	↓ α-syn expression in the lumbal IML of SRT mice ↓ α-syn expression in the ipsilateral DMV of HRT mice ↑ dopaminergic cell death in the ipsilateral SNc of HRT mice ↓ motor deficits (rotarod test)	Pan-Montojo et al. 2012 [26]
SD rats injected with brain lysates from PD patient into the intestine wall of stomach and duodenum	α-syn immunoreactivity in the intestinal wall and in the vagal nerve as well as in ChAT-positive neurons in the DMV (microtubule associated transport)	Holmqvist et al. 2014 [107]
C57 /BL6 mice treated with LPS	↑ α-syn expression in the large intestine p-α-syn in colonic myenteric neurons ↑ intestinal permeability	Kelly et al. 2014 [101]
Aged Fischer rats fed with *E. coli* producing amyloid protein curli	↑ α-syn deposition in gut ganglion cells (myenteric plexus and submucosa) and in neurons in hippocampus and striatum ↑ Iba-1 and GFAP immunoreactivity in the striatum, hippocampus and neocortex	Chen et al. 2016 [100]
**Prion-like spread of α-synuclein**		
Thy-1 α-syn TG mice injected with MCNSCs into the hippocampus	hα-syn immunoreactivity in MCNSCs caspase 3 immunoreactivity in MCNSCs	Desplats et al. 2009 [123]
SD rats with engineered nigral neurons to express hα-syn by AAV2/6 intrastriatally transplanted with embryonic VM neurons	hα-syn immunoreactivity in grafted neurons EEA1-positive transmitted hα-syn PK-sensitive and p-α-syn-negative transmitted hα-syn	Angot et al. 2012 [122]
TgM83 mice inoculated intracerebrally with symptomatic aged M83 brain lysates harbouring aggregated α-syn or PFFs	p-α-syn immunoreactivity in the CNS thioflavin-S- and Ub-positive inclusions in the CNS ↓ TH-positive neurons ↑ GFAP and Iba-1 immunoreactivity in the CNS ↓ survival period	Luk et al. 2012 [116]
Young TgM83 mice inoculated intracerebrally with homogenates of the brain of old TgM83 mice	p-α-syn expression in the brain homogenate p-α-syn immunoreactivity in diffuse perikaryal inclusions and dystrophic neurites paralysis ↓ survival period	Mougenot et al. 2012 [119]
C57BL/6 J mice injected into substantia nigra with recombinant α-synuclein monomer, fibrils or insoluble fraction of brain tissue from DLB patients	p-α-syn immunoreactivity as well as Ub- and p62-positive inclusions in the brain including substantia nigra, amygdala, dentate gyrus, hippocampus, fimbria, stria terminalis, hypothalamus, somatosensory area, visual cortex, cingulate cortex and corpus callosum ↓ enkephalin	Masuda-Suzukake et al. 2013 [112]
C57BL/6 mice intracerebrally inoculated with human LB fraction Rhesus monkeys intrastriatally inoculated with human LB fraction	Mice: p-α-syn immunoreactivity in the striatum and neocortical areas ↓ SNpc TH-positive and Nissl-positive neurons ↑ Iba1-positive cells in the SNpc ↑ impaired motor ability (pole test) Monkey p-α-syn immunoreactivity in the in the putamen, SNpc, globus pallidus, precentral gyrus, superior frontal gyrus, and the entorhinal area in the temporal cortex ↓ SNpc TH-positive neurons ↓ striatal dopaminergic innervation and dopaminergic terminal	Recasens et al. 2014 [120]
SD rats injected with hα-synuclein-AAVs into the vagus nerve, controls	hα-syn immunoreactivity in DMV and nucleus ambiguous, MO, pons and midbrain ↑ rat and total (rat +human) α-syn mRNA vs. controls in MO	Ulusoy et al. 2013 [121]
TgM83 mice injected intracerebrally with brain homogenate from MSA patients	p-α-syn in the striatum, motor cortex, and thalamus ↑ GFAP and Iba-1 immunoreactivity in the brainstem neurologic dysfunction (ataxia, dysmetria, bradykinesia, and circling behaviour) ↓ survival periods	Watts et al. 2013 [118]
M20 and M83Tg mice intracerebrally injected with recombinant amyloidogenic or soluble α-syn	p-α-syn immunoreactivity in the hippocampus, cortex, striatum, midbrain, and brainstem, amygdala, thalamus, and hypothalamus NFL and p62 -reactive inclusions ↑ GFAP and Iba-1 immunoreactivity in the brain	Sacino et al. 2014 [111]
TgM83 TgM20 mice injected intramuscularly with a fibrillar mouse or human α-syn	hind limb paralysis p-α-syn immunoreactivity in the spinal cord, brainstem, midbrain, and cortex (astrocytes and neurons) p62 immunoreactivity in the brain and spinal cord ↑ GFAP and Iba-1 immunoreactivity in the CNS ↓ motor function ↓ survival period	Sacino et al. 2014 [108]
Wistar rats injected with α-syn (oligomers, fibrils or ribbons) into SN or striatum and rAAV-driven α-syn overexpression	p-α-syn immunoreactivity in the whole brain p62-positive inclusions ↓ TH-positive neurons, dopaminergic cells, projecting axons ↓ spontaneous bursts of action potentials in synapses ↑ motor deficit (↓ forelimb contacts in the cylinder test)	Peelaerts et al. 2015 [113]
TgM83 mice injected intraperitoneally or intraglossally with fibrils of human or mouse α-synuclein	p-α-syn immunoreactivity in the brain and spinal cord Sarkosyl-insoluble and Ub- and p62-positive inclusions in the brain and spinal cord ↑ GFAP and Iba-1 immunoreactivity in the brain paralysis, kyphosis, ↓ activity ↓ body weight	Breid et al. 2016 [105]
WT and TgLag3 mice striatally injected with α-syn PFFs	↑ p-α-syn immunoreactivity in the SNpc TH–positive neurons ↓ TH– and Nissl-positive neurons in the SNpc ↓ DA, DOPAC, HVA ↓ TH and DAT ↓ motor function (pole and hindlimb clasping tests)	Mao et al. 2016 [115]
C57BL/6J mice injected with mPFFs or HuPFFs into OB	p-α-syn immunoreactivity within olfactory and brain regions (above 40 brain regions and subregions) ↑ p62-, Ub-, and thioflavin S-positive inclusions ↑ olfactory dysfunction	Rey et al. 2016 [109]
C57BL/6 J and C3H/HeJ mice and SD rats intracranially injected with α-syn fibrils or monomer	p-α-syn immunoreactivity in the SNpc, striatum, amygdala, cortex, and thalamus ↓ TH-positive and Nissl-positive neurons in the SNpc	Abdelmotilib et al. 2017 [114]
Tg A53T;CCK-GFP l mice	α-syn immunoreactivity in EECs of duodenum and colon α-syn immunoreactivity in submucosal enteric nerves TH-positive EECs α-syn in regions of GFAP-positive glial processes	Chandra et al. 2017 [124]
B6/C3H mice injected intraventricularly with rAAV-EV and intrahippocampally with α-syn fibrils	p-α-syn immunoreactivity in the whole brain argyrophilic and p62 immunoreactive inclusions ↑ GFAP and Iba-1 immunoreactivity in the forebrain	Koller et al. 2017 [110]
TgM83 mice injected intraperitoneally or intracerebrally with brains homogenates of sick M83 mice or MSA patients	p-α-syn immunoreactivity in the cerebellum, cerebral cortex, striatum, brainstem, mesencephalon, and in the spinal cord hind limb paralysis, balance disorder ↓ survival periods p-α-syn expression in the brainstem	Sargent et al. 2017 [117]
SD rats injected with hα-synuclein-AAVs into the vagus nerve or the midbrain	hα-syn immunoreactivity in DMV, nodose ganglion, efferent DMV projections and afferent vagal fibers, MO, NTS and gastric wall after vagal injection hα-syn-immunoreactivity in the striatum, hypothalamus, locus coeruleus, MO, DMV neurons, and gastric wall after midbrain injection	Ulusoy et al. 2017 [93]

↑ = increase, ↓ = decrease; AAVs—adeno-associated viral vectors; ASO—Thy1-αSyn (alpha-synuclein-overexpressing); ChAT—choline acetyltransferase; CNS—central nervous system; CP—caudoputamen; DA—dopamine; DAT—dopamine transporter; DLB—dementia with Lewy bodies; DMV—vagal dorsal motor nuclei; DOPAC—3,4-dihydroxyphenylacetic acid; EEA1—early endosome antigen 1; EECs—enteroendocrine cells; ENS—enteric nervous system; FC—frontal cortex; GF—germ-free; GFAP—glial fibrillary acidic protein; hα-syn—human α-synuclein; HRT—hemivagotomized rotenone-treated; HuPFFs—WT human α-syn preformed fibrils; HVA—homovanillic acid; Iba-1—ionized calcium-binding adapter molecule 1; IL-6—Interleukin 6; IML—intermediolateral column of the spinal cord; iNOS—inducible nitric oxide synthase; LBs—Lewy bodies, LNs—Lewy neuritis; MCNSCs—mouse cortical neuronal stem cells; MHC-II major histocompatibility complex class 2; Mid—midbrain; MO—medulla oblongata; mPFFs—WT mouse α-syn PFFs; MSA—multiple system atrophy; NFL—low-molecular-mass neurofilament subunit; NTS—nucleus of the tractus solitaries; OB—olfactory bulb; PD—Parkinson’s disease, PFFs pre-formed fibrillar assemblies of recombinant α-synuclein; PK—proteinase K; SCFAs—short-chain fatty acids; SD—Sprague-Dawley; SN—substantia nigra; SNpc—substantia nigra pars compacta; SPF—antibiotic-treated specific pathogen-free; SRT—sympathectomized rotenone-treated; TH—tyrosine hydroxylase; TLR2—toll-like receptor 2; TNF-α—tumor necrosis factor, VM—ventral mesencephalon; p-α-syn—phosphorylated α-synuclein, p62—nucleoporin protein; Ub—ubiquitin, TG –transgenic, WGTT—whole-gut transit time; WT—wild type.

## Abbreviations

CNS	Central nervous system
DMV	Dorsal motor nucleus of the vagus
EECs	Enteroendocrine cells
ENS	Enteric nervous system
GI	Gastrointestinal
IML	Intermediolateral cell column
KYN	Kynurenine
LAG3	Lymphocyte-activation gene 3
LBs	Lewy bodies
LNs	Lewy neuritis
LP	Lewy pathology
LPS	Lipopolysaccharide
PANS	Peripheral autonomic nervous system
PFFs	Preformed fibrillar α-synuclein assemblies
PD	Parkinson’s disease
SCFAs	Short chain fatty acids
SIBO	Small intestinal overgrowth
SNpc	Substantia nigra pars compacta
TNTs	Tunneling nanotubes
VM	Ventral mesencephalic
